# Usage of Near-Infrared Spectroscopy for Inline Monitoring the Degree of Curing in RTM Processes

**DOI:** 10.3390/polym13183145

**Published:** 2021-09-17

**Authors:** Moritz Salzmann, Yannick Blößl, Andrea Todorovic, Ralf Schledjewski

**Affiliations:** 1 Department of Polymer Engineering, Processing of Composites, Montanuniversität Leoben, 8020 Leoben, Austria; yannick.bloessl@gmx.de (Y.B.); Ralf.Schledjewski@unileoben.ac.at (R.S.); 2 Department of Polymer Engineering, Material Science and Testing of Polymers, Montanuniversität Leoben, 8020 Leoben, Austria; andrea.todorovic@unileoben.ac.at

**Keywords:** near-infrared spectroscopy (NIR), epoxy-resin, inline monitoring, resin transfer molding (RTM)

## Abstract

Near-infrared spectroscopy (NIR) was implemented in the resin transfer molding (RTM) process to inline monitor the degree of curing of a bio-based epoxy resin, which consists of epoxidized linseed oil (resin) and citric acid (hardener), respectively. A NIR micro-spectrometer was used for the development of robust calibration models using partial least squares (PLS) regression. Since the micro-spectrometer offers a smaller wavelength range compared with conventional NIR devices, and typical absorbance peaks are not directly involved in the captured data range, the results show new insights for the utilization of this technology. Different pre-treatments of the spectroscopic data have been tested, starting with different reference spectra, i.e., uncured resin and polytetrafluorethylene (PTFE), and followed by chemometrical algorithms. As a reference method for the degree of curing, direct current (DC) supported by differential scanning calorimetry (DSC) was used. The results show the potential of these cost-efficient and compact NIR micro-spectrometers for the intended inline monitoring purpose to gain relevant information feedback during the process.

## 1. Introduction

Fiber-reinforced polymer composites (FRPC) offer the possibility to manufacture optimized lightweight components. Depending on the processing technique used, structure optimization might include locally adapted reinforcements. Liquid composite molding (LCM) techniques, such as resin transfer molding (RTM) or vacuum infusion, represent a group of manufacturing processes enabling one to produce a wide range of different types of FRPCs, from small to extremely large sizes, from simple to even complex-shaped geometry and incorporating additional reinforcements. All LCM procedures consist of the preparation of the dry reinforcing structure, typically called preform, infiltration by a low viscosity resin system and curing the fully impregnated FRPC [1]. Since easy prototype manufacturing, as well as highly automated series production, is possible, LCM processing is widely used. Especially for series production, process optimization [2] and reproducibility are gaining importance [3]. With increased automatization in the field of processing composites, efficient inline monitoring techniques are required to ensure process stability and quality. Several promising techniques have been identified [4] and some are now in the focus of the research community, e.g., ultrasonic (US), dielectric analysis (DEA) or direct current (DC).

One technology hardly tested in the field of processing composites so far is near-infrared spectroscopy (NIR). NIR uses light in the region of near-infrared from wavelengths of 700–2500 nm, which activates overtone and combination vibrations of molecules [5]. Together with a previously created calibration, the resulting spectra can be used to determine properties of the sample. The use of optical fibers enables inline measurements by NIR, as the spectrometer can be deployed outside the process. By one (or several) optical fiber(s), the light is transmitted into the process and a further optical fiber guides the reflected or transmitted light to the spectrometer. A substantial advantage of NIR is the possibility to determine several parameters with just one measurement, as spectra can contain information of many parameters at once [6,7], e.g., the resin content, volatile content and degree of curing in the prepreg production [8]. In other industries, such as pharmacy [9,10,11] and food production [12,13], NIR has been occasionally applied for inline monitoring.

While NIR is well established in laboratories for investigating curing kinetics, e.g., [14,15,16], it is used for inline monitoring production processes of FRPC to a limited extent. The only case exploiting NIR for inline monitoring in composite processing represents its usage in prepreg production. For different parameters along the processing route, such as resin and volatile content of the prepreg, robust partial least squares (PLS) regression models have been developed [6,7,8,17,18,19,20]. For other processes in the field of composites, NIR has just been examined to determine the moisture content of a flax fiber textile inline in the RTM process. Here, a good correlation between the H_2_O-Peak at 1924 nm and the moisture content was found [21].

In recent years, NIR micro-spectrometers [22] have been developed. Their low price compared with traditional NIR spectrometers makes them interesting for further applications. On the other hand, they are technically limited in comparison with traditional spectrometers, either in resolution or in the covered wavelength range.

Due to the limited covered wavelength, range-relevant absorbance peaks might not be within the measuring range of such a spectrometer. If several parameters are to be monitored, it might be necessary to use a certain spectrometer, although it is measuring in a wavelength range without any absorbance peak directly from the sample.

FRPC based on renewable materials are gaining more and more interest [23]. Beside natural fibers as reinforcement, resin systems with an extremely high bio-based carbon content are especially under development [24]. The present study expands previous work determining moisture content of flax fiber in the RTM process [21], using the same NIR micro-spectrometer. As there are no relevant epoxy peaks (1379 nm and 2353 nm) [25] in the measuring range of the spectrometer, the question arises of whether the available changes in the spectra are useful to determine the degree of curing. The aim of the present study is the inline monitoring of the degree of curing of epoxidized linseed oil (ELSO) with citric acid (CA) as hardener [23]. To do so, an NIR-reflectance probe was installed within the RTM tool. Afterwards, different calibration models to determine the degree of curing were developed by using different reference spectra, spectral pretreatments and PLS regression. Subsequently the quality of the models was validated, based on the number of leverage variables (LV), coefficient determination (R²), the root mean square error of cross validation (RMSECV) and the root mean square error of prediction (RMSEP).

## 2. Materials and Methods

In the RTM process, the degree of curing of the ELSO/CA formulation was monitored inline by NIR. As reference measurement method for the PLS regression models direct-current (DC) supported by differential scanning calorimetry (DSC) was chosen.

### 2.1. Materials

As matrix, an epoxidized linseed oil (ELSO) provided by Kompetenzzentrum Holz GmbH (Austria) was used. Information about the synthesis can be found in the study from Anusic et al. [26]. As a hardener, crystalline citric acid (CA), having a purity of 99.5% (Carl Roth GmbH+Co. KG, Karlsruhe, Germany), was used. CA was milled in a self-constructed ball mill for 24 h. For further preparation of the matrix system, 1000 g ELSO, 252.5 g CA and 6.25 g (0.5 wt.%) deaerator (Epinal EL 12.42, bto epoxy GmbH, Amstetten, Austria) were dispersed in a dissolver for 20 min with 6000 rpm. Afterwards, it was further dispersed for 3 h with a bead mill (Dispermat SL12, VMA Getzmann GmbH, Reichshof, Germany) with up to 2000 rpm. The dispersed ELSO/CA-mixture was stored at around 0 °C until processing.

### 2.2. RTM Process

**Figure 1:**
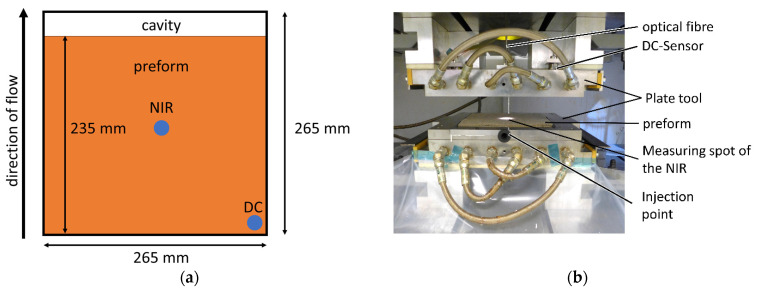
(**a**) Positions of the NIR probe, in the center of the plate, and DC sensor in the lower right corner. (**b**) opened RTM plate mold.

A rectangular RTM mold (265 × 265 × 4 mm^3^) was used for the test series. The mold was preheated to 50 °C. Five layers of a flax fiber textile (Biotex Flax 400 g/m^2^ 2 × 2 Twill, 265 × 235 mm^2^, Composites evolution ltd., Chesterfield, United Kingdom) were dried at 120 °C for 2 h and placed, flush with the front edge, in the mold before closing. A total of 648 g ELSO/CA was defrosted and a further 3.24 g Epinal EL 12.42 were added, increasing the amount of deaerator to 1 wt.%. The resin was degassed for 20 min. Afterwards, the resin was placed in a pressure pot for injection. The resin was injected with an air pressure of 4 bar. After the injection was stopped, the temperature of the mold was increased from 50 °C to 80 °C within 20 min and the resin was cured for 24 h at 80 °C. After 24 h the plate was demolded. The RTM mold and positions of the used inline sensors on the plate are shown in Figure 1.

### 2.3. Inline Measurements

The NIR probe, used in reflection mode, as well as the DC sensor, were integrated into the RTM mold, allowing inline measurements during the experiment.

Light from a halogen light source (AvaLight-HAL-S-Mini, Avantes BV, Appeldorn, Netherlands) was guided by the NIR probe (400 µm fiber core, Avantes BV, Appeldorn, Netherlands) into the mold. To protect the probe from the resin, a 5 mm borosilicate glass panel (Schott AG, Mainz, Germany) was installed. The reflected light was guided by a S2.2 NIR micro-spectrometer (Spectral Engines GmbH, Steinach, Germany). The spectrometer measures at a wavelength range from 1650 to 2150 nm with 2 nm steps. Every minute one spectrum was saved. Each saved spectrum was the average of 5 spectra, which in turn consisted of averaging 10 measurements at each wavelength.

The DC sensor (Synthesites Innovative Technologies Ltd., Piraeus, Greece) had direct contact with the cavity and was set to a measuring frequency of 1 Hz.

### 2.4. DSC Measurements

The reaction enthalpy of the uncured resin formulation (ELSO/CA), of the partially cured resin and of the cured resin was determined by DSC (DSC1, Mettler Toledo GmbH, Schwerzenbach, Switzerland). An overview of the sample preparation is given in Table 1. Thermograms of the ELSO/CA samples were recorded in the temperature range between 25 °C and 240 °C, with a heating rate of 10 K/min. Measurements were performed under nitrogen atmosphere (gas flow = 50 mL/min) in 40 µL aluminum crucibles, and the sample mass was 10 ± 1 mg. The instrument was calibrated using the melting enthalpy and the onset temperatures of the melting of indium standards. The evaluations were done according to ISO 11357-5 (curing enthalpy) by using the Mettler Toledo DSC software (STARe, Mettler Toledo GmbH, Schwerzenbach, Switzerland). The number of replicate measurements was two.

To prepare the partially cured sample (sample 2), a small amount of resin was placed in the preheated RTM mold (50 °C). The mold was closed and heated to 80 °C within 20 min. The sample was taken out and cooled by air convection to room temperature. Afterwards, the DSC measurement was performed, as described above.

### 2.5. Data Analysis

Inline NIR spectra are measured during the production process while the corresponding reference values can be also captured in the process or via preparatory measurements on a laboratory setup, depending on the requirements of the reference methods. Which reference methods are chosen depends on the monitored parameters. In the case of this study, DC and DSC were used as reference methods to determine the degree of curing. Each spectrum was assigned to a reference value. Afterwards, the data was divided into a calibration and a validation data set.

Before using a multivariate regression model, the data are preprocessed. The preprocessing is to strengthen the influence of the wanted information on the spectra and reduce noise. There are two steps in the preprocessing. First a suitable reference spectrum is chosen. This is typically a spectrum from the monitored process or of a reference material, e.g., polytetrafluoroethylene (PTFE). Second mathematical techniques, called chemometrics, are applied. To find the optimal preprocessing is nearly impossible due the amount of chemometric algorithms. Therefore, four common chemometric algorithms were used in this publication on its own, without the aim to find an optimal preprocessing. This allows the influence of each algorithms on the results to be seen. The used algorithms were mean centering, 1st derivative (1st Deriv.), multiplicative scattering (MSC) and smoothing by applying a Savitzky Golay filter. Using mean centering an average spectrum of the calibration data set is subtracted from each spectrum. The center of the data becomes the new origin, which simplifies methods of the multivariate data analysis. The 1st Deriv. removes shifts of absorbance and can minimize random noise and narrow peaks in spectra. Commonly, a smoothing is applied afterwards. MSC reduces the effect of light scattering on the spectra. Savitzky Golay uses local polynomial regression to reduce spikes in the spectra. For this study, a window length for the Savitzky Golay of 15 was used.

Afterwards, the regression model was tested. Multivariate regression models were reducing the number of required variables to describe a data set. In terms of NIR, each measured wavelength represents one variable. As a spectrum consists of measurements at up to several hundred wavelengths, it is not practicable to look at the absorbance at every single wavelength. Nor is it necessary as, e.g., a peak in a spectrum is affecting the absorbance at several wavelengths in a similar way. These similarities are used by multivariate regression models to reduce the number of required variables. There are different multivariate regression models, such as principal component regression (PCR) or multi linear regression (MLP). The most common one is the partial least squares regression (PLS). For PLS, the new variables are called leverage variables (LV).

It is important to mention that the suitability of a preprocessing can be evaluated only after the regression modelling. Afterwards, the regression model is tested with the validation data.

Pretreatment of the data and creating the partial least squares (PLS) regression were performed according to [27] with the MatLab-based PLS toolbox (Eigenvector Research Inc., United States). To develop the PLS model, 130 NIR measurements were used for calibration and 45 for prediction, respectively. The coefficient of determination (R²), the root mean square error of cross validation (RMSECV) and the root mean square error of prediction (RMSEP) were used to compare different models and identifying influences of used chemometrics.

## 3. Results

### 3.1. Reference Measurements

**Figure 2:**
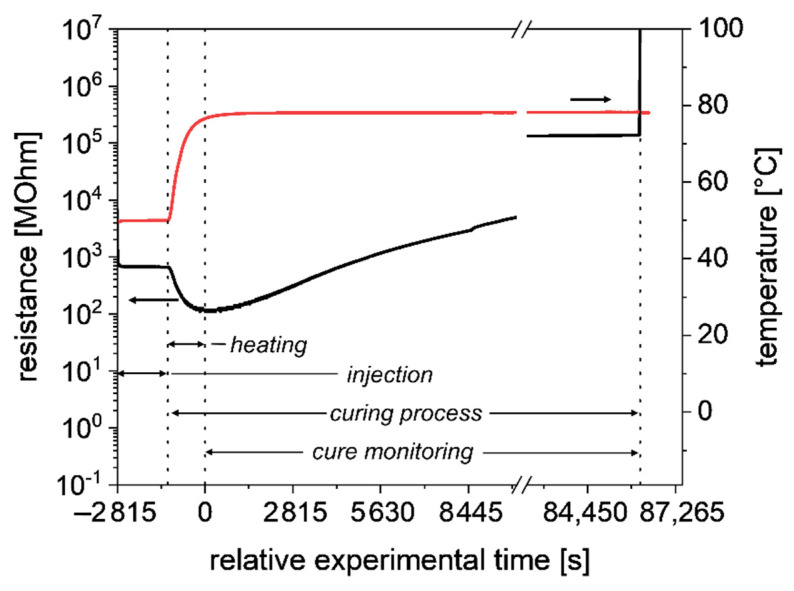
DC (black)-and temperature (red) data of the RTM experiment. Due to the increase in temperature the resistance first drops before it increases due to increasing degree of curing. Only the part with increasing resistance of the curing process can be used to reference the NIR spectra.

As reference methods DC, supported by DSC, were chosen. DC allowed the measurement of the electrical resistance in direct contact with the thermoset resin system, providing the relevant data of the degree of curing [28]. In Figure 2 DC data of the experiment are shown. The relative experimental time is set to 0 s, from the time when the DC data can be used for the cure monitoring. The starting point, at −2815 s, marks the arrival of the injected resin at the sensor position. After 1625 s, at −1190 s, the injection is finished and the curing process started. During the curing process the resistance first decreased before increasing. The decrease was a result of the increasing temperature. Increasing temperature decreases viscosity, which leads to a better ion conductivity. A low viscosity allows a better movement of ions, thus resistance decreases. With increasing degree of curing, the ion conductivity decreases and resistance increases.

For isothermal experiments, DC measurements for the degree of curing, α, can be calculated with the following equation [28].

(1) $$\alpha \left(t\right)=\frac{\log\left(\Omega_{t}\right)-\log\left(\Omega_{0}\right)}{\log\left(\Omega_{\infty }\right)-\log\left(\Omega_{0}\right)}\times \alpha_{\infty }$$

where $\Omega_{t}$ is the resistance at the time *t*, $\Omega_{0}$ is the resistance at the beginning of the curing process, $\Omega_{\infty }$ is the resistance at the end of the curing process and $\alpha_{\infty }$ is the degree of curing at the end of the curing process. Since Equation (1) is limited to isothermal curing processes, it cannot be applied directly on the experimental data. The decrease in resistance during the heating phase has to be cut out, as it results from the heating. However, the curing during the heating phase has still to be considered. This results in a modified formula for α, s. Equation (2)

(2) $$\alpha \left(t\right)=\alpha_{1}+\frac{\log\left(\Omega_{t}\right)-\log\left(\Omega_{1}\right)}{\log\left(\Omega_{\infty }\right)-\log\left(\Omega_{1}\right)}\times (\alpha_{\infty }-\alpha_{1})$$

where $\alpha_{1}$ is the degree of curing at the beginning of the observed curing phase and $\Omega_{1}$ is the corresponding resistance. To obtain α(t) of the isothermal phase the minimum of the resistance at 0 s is defined as $\Omega_{1}$.

**Figure 3:**
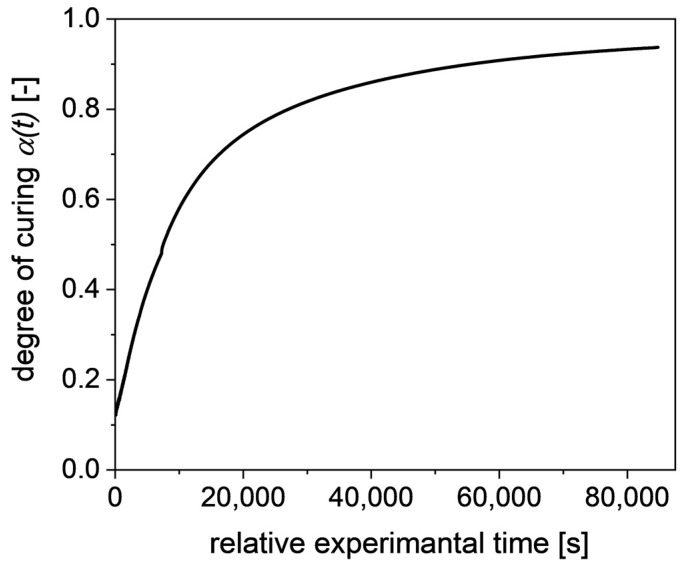
Degree of curing α(t) at 80 °C over time, used as reference curve for the NIR data.

$\alpha_{1}\;$ and $\alpha_{\infty }$ are determined by DSC, using Equation (3).

(3) $$\alpha \left(t\right)=\frac{H_{part}}{H_{tot}}$$

where $H_{part}$ is the remaining reaction enthalpy of a sample cured for the time t, and $H_{tot}$ is the reaction enthalpy of an uncured sample. For $\;\alpha_{1}$ and $\alpha_{\infty }$, a degree of curing of 12.83%  and 93.74% is obtained, respectively. The resulting graph of the degree of curing is shown in Figure 3. 

### 3.2. NIR Data

The used spectrometer (NIRONE S2.2) is based on a Fabry–Perot interferometer. It is measuring the intensity of light at each wavelength coming from or through the sample. The intensity contains information of all molecules within the measuring volume, relevant as well as irrelevant information.

**Figure 4:**
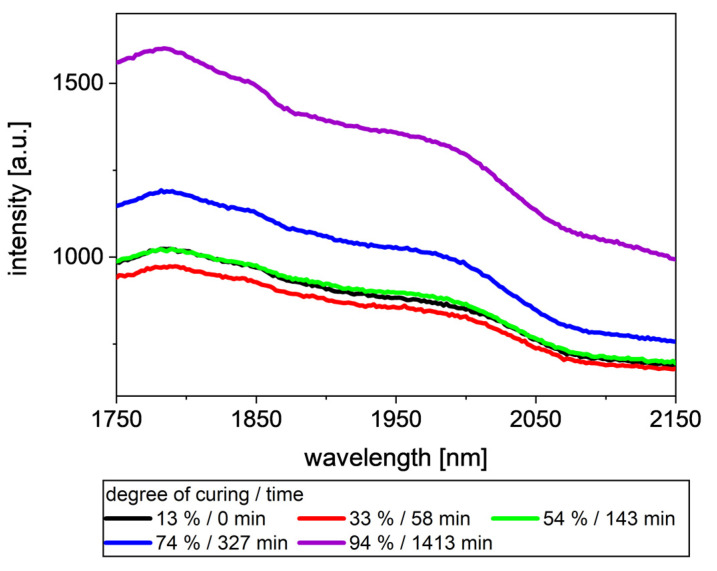
Intensities at different degrees of curing of the ELSO/CA resin. Intensities increase with increasing degree of curing. Additionally, a step at 1850 nm occurs with increasing degree of curing.

In Figure 4, intensities between 1750 nm and 2150 nm at different degrees of curing are shown. With an increasing degree of curing, the baseline of the intensity data first decreases slightly before it recovers. Comparing the 13% degrees of curing and 54%, it is evident that changes in the measured intensity occurs mainly between 1850 nm and 2020 nm. Above 54%, the baseline is shifting upwards. Additionally, a step occurs at around 1850 nm. The decrease and later increase in the intensity could be an artefact of the measuring series, as it contains data of only one RTM shot. In our opinion, it is more likely the result of the decreasing particle size of CA [29] and a changing color of the composite [30], both caused by the curing reaction. As it is not the topic of this study, the reason for this change of trend is not further investigated.

**Figure 5:**
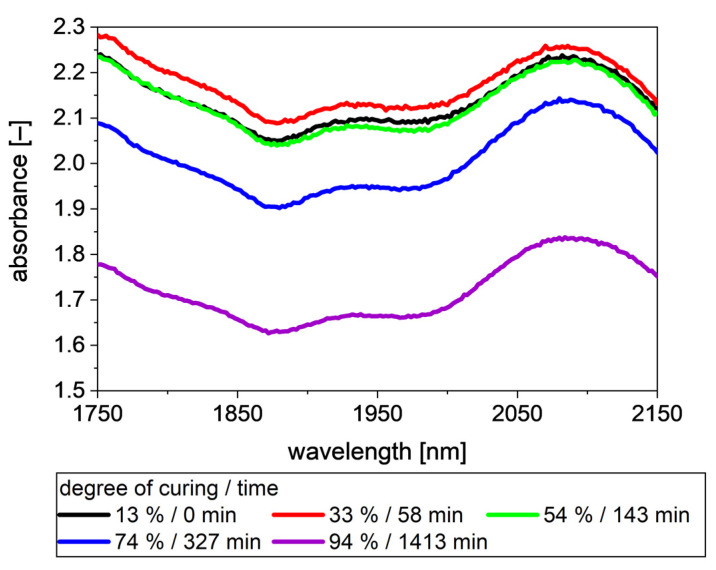
Spectra at different degrees of curing with PTFE as reference spectrum. Similar to Figure 4, mainly a shift of the baseline is observable, as no peaks of the involved functional groups occur within the measured wavelengths.

**Figure 6:**
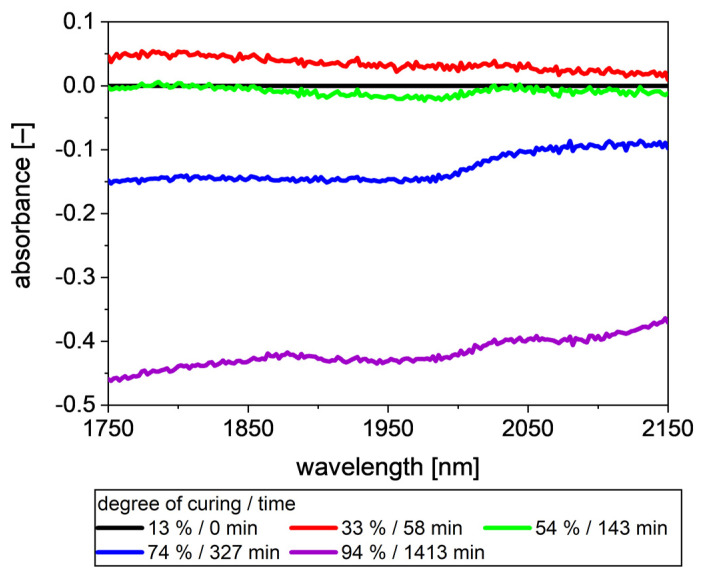
Spectra at different degrees of curing, using the spectrum of uncured resin as reference spectrum and not PTFE as shown in Figure 5, the baseline of the absorbance decreases. Besides the shift of the baseline, more and more steps at different wavelengths occur.

To overcome the problem that the intensity data includes a bunch of unwanted data, the absorbance is calculated. The absorbance is calculated as an analogue to the law of Beer–Lambert, described in Equation (4):

(4) $$A=-\log\left(\frac{I}{I_{0}}\right)$$

where A is the absorbance, I is the measured spectrum and $I_{0}$ is a reference spectrum. In the absorbance, only the information which is not included in the reference spectrum remains. In the present study, polytetrafluoroethylene (PTFE), s. Figure 5, and the first spectrum of the monitored curing process, s. Figure 6, are used as reference spectra.

For analytical measurements, typically a standard material, e.g., polytetrafluoroethylene (PTFE), is used as reference. This results in the spectra are typically known from spectroscopic measurements. Figure 5 shows the absorbance at different degrees of curing calculated with a PTFE spectrum as reference. The spectra show the opposite trend than the intensity data. The absorbance first increases before it decreases. The decrease rises with increasing degree of curing. The small occurring changes in the spectra are well visible between 13% and 54% degrees of curing. Between 1850 nm and 2020 nm, the absorbance of 54% is slightly lower than that of 13%. In the rest of the observed wavelength ranges, both spectra show nearly identical absorbance. The “peak” at around 1930 nm narrows at 54% degrees of curing, resulting in larger difference to the spectrum of 13% degrees of curing between 1930 nm and 2020 nm than between 1850 nm and 1930 nm. 

Instead of PTFE as a reference spectrum, a spectrum from the process can also be used. In this case, only changes during the process remain in the spectra. Figure 6 shows spectra as shown in Figure 5 with the first spectrum, measured at a degree of curing at 13% as the reference spectrum. In the following this spectrum is referred as “uncured resin” (UR), as it is the spectrum with the lowest degree of curing used in this study. The negative absorbance results from the increasing intensity in the later phases of the curing process. Using the first spectrum as a reference spectrum, according to Equation (4), the resulting absorbance is negative.

The spectra show the same trend as the spectra with PTFE as a reference (Figure 5), with first an increase in absorbance and with an increasing degree of curing than a decrease in absorbance. Comparing the spectra of 13% and 54% degrees of curing, they differ again between 1850 nm and 2020 nm. Additionally, 54% degrees of curing show a slightly lower absorbance above 2060 nm. Much more clearly compared with the intensity and PTFE reference spectra, changes with increasing degree of curing at higher degrees of curing are visible. The spectrum of 74% degrees of curing shows a step at 2016 nm, whereas in the spectrum of 94% degrees of curing, the step is shifted to 2006 nm. Further, the absorbance increases between 1750 nm and 1880 nm and above 2100 nm.

### 3.3. PLS Model for the Degree of Curing

The observed range of the degree of curing for the calibration set is between 13% and 94%. In order to obtain a good regression model, the influence of reference spectrum, PTFE and uncured resin on the PLS regression is tested without any pretreatment. The results are summarized in Table 2. When using the uncured resin as reference spectrum (PLS model UR) with two leverage variables (LV), a regression model already with extremely good prediction properties is obtained.

**Figure 7:**
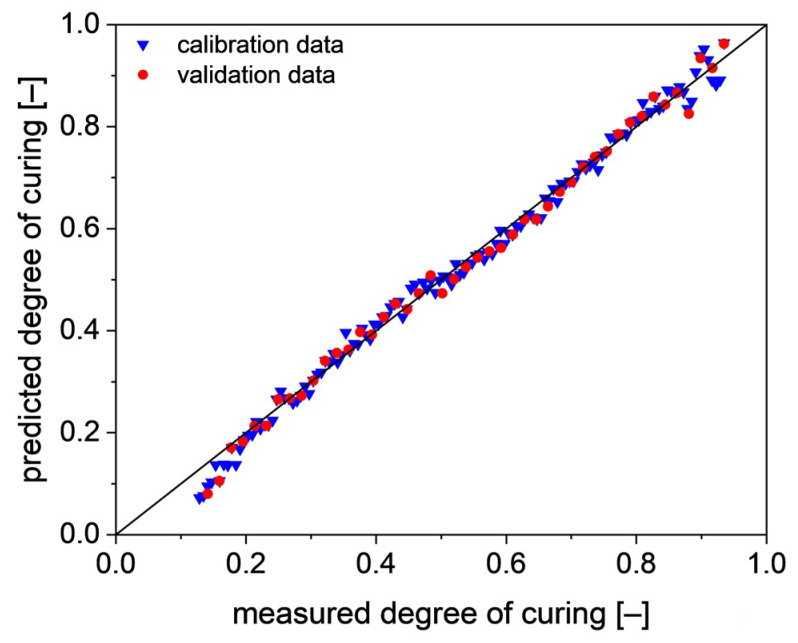
Correlation between the predicted values and measured values for the degree of curing, using the uncured resin as reference spectrum. (PLS model UR).

The corresponding correlation between the measured and predicted values of the degree of curing is shown in Figure 7. The predicted degree of curing largely agrees with the measured degree of curing. Only at the borders of the model are there small deviations. At the beginning of the curing process, UR underestimates the degree of curing, whereas above 85% curing, the predicted values start scatter slightly.

Using PTFE as the reference spectrum (PLS model PTFE-B), four LV are required to obtain PLS model with similar values as UR regarding *R*^2^, RMSECV and RMSEP. As an increasing number in LVs also increases the statistical uncertainty, a PLS model with PTFE as the reference spectrum and two LV is developed (PLS model PTFE-A). With just two LV, the obtained *R*^2^, RMSECV and RMSEP are significantly worse than the values of PTFE-A.

**Figure 8:**
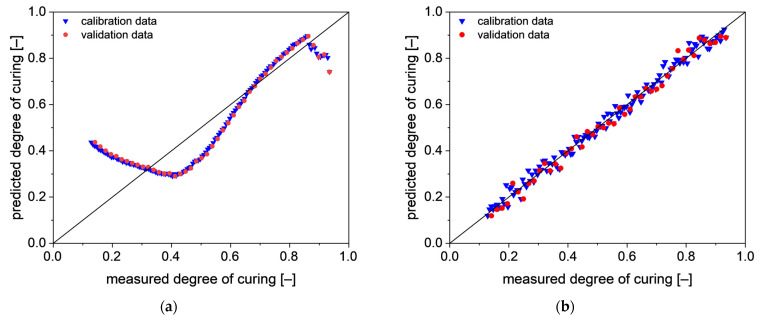
Correlation between the predicted values and measured values for the degree of curing, using the PTFE as reference spectrum (**a**) with 2 LV (PTFE-A) and (**b**) with 4 LV (PTFE-B).

Plotting the predicted degree of curing versus the measured degree of curing shows the same result (s. Figure 8). For PTFE-A, the predicted degree of curing does not match the measured degree of curing at all. Up to around 60% degrees of curing, the predicted values are not correlated in any way with the measured ones. Above 70% degrees of curing, the predicted values slightly overestimate the degree of curing. At around 90%, PTFE-A starts to scatter much more than before and underestimates the degree of curing more and more clearly with increasing degree of curing.

Using four LV to predict the degree of curing of spectra with PTFE as reference results in a PLS model with good prediction qualities. Compared with UR, the scattering above the whole range of the degree of curing seems to be larger. In return, the scattering does not seem to increase towards the model boundaries.

As four LV are still not many LV for a PLS model compared with other PLS regressions [8,17], different preprocessings are tested on the data set using uncured resin and PTFE as reference spectra. The results are shown in Table 3 and Table 4. Both data sets are pretreated with different chemometric algorithms, mean center, 1st Derivative (1st Deriv.), multiplicative scattering (MSC) and smoothing by Savitzky Golay.

Compared with UR, using mean center (UR-1) and 1st Deriv. (UR-2) as preprocessing methods, three LV are required, instead of two, to obtain comparable values in *R*^2^, RMSECV and RMSEP. UR-1 improves in *R*^2^, RMSECV and RMSEP, whereas UR-2 has, despite the addition of LV, worse values than UR. Smoothing (UR-4) weakens the quality of the model slightly by reducing *R*^2^ and increasing in RMSECV and RMSEP. Notable is UR-3, which is preprocessed with MSC; with a *R*^2^ = 0.088 a regression model is received with no capability of predicting a degree of curing. That RMSECV with 0.25 and RMSEP=0.23 are one order of magnitude lager than in the other models, thus not further relevant as UR-3 has no capability for predicting the degree of curing. The reason is discussed in a later section of this paper.

In Table 4, PLS models with PTFE as the reference spectrum using different preprocessings are listed. Compared with the UR-based models, the influence of the preprocessing methods differs. The models obtained with 1st Deriv. (PTFE-2) and smoothing (PTFE-4) require just as many LV as PTFE-B with 4 LV. With a difference in RMSECV of 0.003 and equal values for *R*^2^ and RMSEP, PTFE-4 can been equal to PTFE-B regarding the model quality, whereas PTFE-2 is slightly worse than PTFE-B in all three parameters.

Using mean center (PTFE-1) and MSC (PTFE-3) has positive effects on the model quality. Both preprocessings allow reduce the amount of LV by one, while increasing *R*^2^, RMSECV and RMSEP slightly. Using PTFEas the reference spectrum, with *R*^2^ = 0.995, RMSECV=0.018 and RMSEP=0.019, the best regression model found is PTFE-1.

## 4. Discussion

The choice of the reference spectrum as well as further preprocessing is affecting the PLS regression to determine the degree of cure of a bio-based ESLO-CA resin during curing in the RTM process. The choice of the reference spectra seems to have the larger impact on the model quality. Using uncured resin as reference spectrum (UR) results in the PLS model requiring 2 LV to predict the degree of curing quite accurately with *R*^2^ = 0.992. To obtain a PLS model with similar qualities using PTFE as reference spectrum, 4 LV are required (PTFE-B). This shows clearly the advantage of using a spectrum directly from the process as a reference spectrum, instead of a spectrum from a reference material. Compared with other studies using NIR for inline monitoring in the field of processing composites, both models require less LV while showing better values regarding *R*^2^, RMSECV and RMSEP [6,7]. The static measuring environment of the RTM process has here some advantages over the referenced prepreg process, as interfering effects such as dirt and changing measuring environment are excluded.

For UR, no preprocessing was found to improve the PLS model further. As only the information about the changes due to the curing process remain in the data, there are no interfering effects anymore, making further processing unnecessary. This is probably also the reason why UR-3, using MSC as preprocessing, does not have any capability of predicting the degree of curing. MSC is designed to eliminate influence of wavelength-dependent scattering effects. However, all these effects have already been eliminated by the transformation to the absorbance data as the reference spectrum is from the same data set as the rest of the used spectra. As shifts of absorbance are often caused by differences in the wavelength-dependent scattering effects and the absorbance changes during the curing process, not enough information remains after applying MSC to predict the degree of curing. This effect could be caused by the design of the experiment as all spectra are from one curing process. If MSC would show the same effects if the reference spectrum was from another run of the experiment is unclear by now.

That the shift in absorbance is not the only important information about the degree of curing shows UR-2. The 1st derivative eliminates the information about the absorbance shift. However, a PLS model is still obtained, able to predict the degree of curing of ELSO-CA and showing that the small changes occurring in the spectra over the curing process are also relevant for the prediction model.

The difference in wavelength-dependent scattering between the reference spectrum and the measured spectra affects the PLS models. The use of MSC PTFE-3 is improving results, requiring one LV less than PTFE-B and obtaining better values regarding *R*^2^, RMSECV and RMSEP.

Between the PLS models based on UR and PTFE-B no trend due to the preprocessing methods can be seen. Only smoothing leads to models of similar quality (UR-4 and PTFE-4) compared with the unprocessed ones. This is expected as the absorbance data show no strong scattering (s. Figure 7 and Figure 8).

The missing of a clear trend of improving the model quality due to a certain preprocessing method together with the results of UR-3 show once more that it is difficult to define fixed rules on how to preprocess spectroscopic data, and that for each data set a suitable preprocessing method has to be found by testing different attempts [27,31].

## 5. Conclusions

The NIR micro-spectrometers used have a limited wavelength range (1750–2150 nm). No absorbance peak, directly affected by the curing reaction of an epoxidized linseed oil (ELSO) with citric acid (CA), was found within the measuring range. Still, valid prediction models could be established using partial least squares regression (PLS). The influence of different reference spectra, namely PTFE and uncured resin (UR), was investigated. For both used reference spectra, robust PLS models were obtained without further preprocessing. Applying different preprocessing on the UR data set did not show an improvement in coefficient of determination (R²), the root mean square error of cross validation (RMSECV) and the root mean square error of prediction (RMSEP), whereas the PTFE data set mean center and multiplicative scattering (MSC) reduced the number of leverage variables to three while improving R², RMSECV and RMSEP.

With the robust prediction models found for the degree of curing NIR, there is a notable inline monitoring technique when processing reactive resin, not only for the resin transfer molding (RTM) process.

## Figures and Tables

**Table 1:** Preparation of the DSC samples.

Sample Number	Resin State	Sample Preparation
1	Uncured	None
2	Partially cured	Heating from 50 °C to 80 °C within 20 min
3	Cured	Cured at 80 °C for 24 h

**Table 2:** Effect of the reference spectra on the PLS.

Abbreviation PLS Model	Reference Spectrum	No. of LVs	*R* ^2^	RMSECV	RMSEP
UR	Uncured Resin	2	0.992	0.024	0.022
PTFE-A	PTFE	2	0.776	0.108	0.111
PTFE-B	PTFE	4	0.989	0.024	0.027

**Table 3:** Different preprocessings for PLS models based on uncured resin as reference spectrum.

Abbreviation PLS Model	Processing	No. of LVs	*R* ^2^	RMSECV	RMSEP
UR	None	2	0.992	0.024	0.022
UR-1	Mean Center	3	0.994	0.018	0.019
UR-2	1st Deriv	3	0.976	0.038	0.037
UR-3	MSC	2	0.088	0.25	0.23
UR-4	Smoothing (SavGol)	2	0.986	0.038	0.036

**Table 4:** Different preprocessings for PLS models based on PTFE as reference spectrum.

Abbreviation PLS Model	Preprocessing	No. of LVs	*R* ^2^	RMSECV	RMSEP
PTFE-B	None	4	0.989	0.029	0.027
PTFE-1	Mean Center	3	0.995	0.018	0.019
PTFE-2	1st Deriv	4	0.967	0.046	0.043
PTFE-3	MSC	3	0.991	0.026	0.024
PTFE-4	Smoothing (SavGol)	4	0.989	0.026	0.027

## Data Availability

The data presented in this study are available on reasonable request from the corresponding author.
